# Evaluation of radixact motion synchrony for 3D respiratory motion: Modeling accuracy and dosimetric fidelity

**DOI:** 10.1002/acm2.12978

**Published:** 2020-07-21

**Authors:** William S. Ferris, Michael W. Kissick, John E. Bayouth, Wesley S. Culberson, Jennifer B. Smilowitz

**Affiliations:** ^1^ Department of Medical Physics School of Medicine and Public Health University of Wisconsin‐Madison Madison WI USA; ^2^ Accuray Incorporated Madison WI USA; ^3^ Department of Human Oncology School of Medicine and Public Health University of Wisconsin‐Madison Madison WI USA

**Keywords:** intrafraction motion, radixact, synchrony, tomotherapy, tracking

## Abstract

The Radixact® linear accelerator contains the motion Synchrony system, which tracks and compensates for intrafraction patient motion. For respiratory motion, the system models the motion of the target and synchronizes the delivery of radiation with this motion using the jaws and multi‐leaf collimators (MLCs). It was the purpose of this work to determine the ability of the Synchrony system to track and compensate for different phantom motions using a delivery quality assurance (DQA) workflow. Thirteen helical plans were created on static datasets from liver, lung, and pancreas subjects. Dose distributions were measured using a Delta4® Phantom+ mounted on a Hexamotion® stage for the following three case scenarios for each plan: (a) no phantom motion and no Synchrony (M0S0), (b) phantom motion and no Synchrony (M1S0), and (c) phantom motion with Synchrony (M1S1). The LEDs were placed on the Phantom+ for the 13 patient cases and were placed on a separate one‐dimensional surrogate stage for additional studies to investigate the effect of separate target and surrogate motion. The root‐mean‐square (RMS) error between the Synchrony‐modeled positions and the programmed phantom positions was <1.5 mm for all Synchrony deliveries with the LEDs on the Phantom+. The tracking errors increased slightly when the LEDs were placed on the surrogate stage but were similar to tracking errors observed for other motion tracking systems such as CyberKnife Synchrony. One‐dimensional profiles indicate the effects of motion interplay and dose blurring present in several of the M1S0 plans that are not present in the M1S1 plans. All 13 of the M1S1 measured doses had gamma pass rates (3%/2 mm/10%T) compared to the planned dose > 90%. Only two of the M1S0 measured doses had gamma pass rates > 90%. Motion Synchrony offers a potential alternative to the current, ITV‐based motion management strategy for helical tomotherapy deliveries.

## 1 INTRODUCTION

Intrafraction motion limits the conformity of radiation therapy treatments by prohibiting tight margins around the clinical target volume (CTV). Without compensating for motion during treatment, sufficiently large planning target volumes (PTV) are necessary to minimize the risk of underdosing the target while also resulting in a larger volume of normal tissue irradiated. This expanded volume is commonly referred to as the internal target volume (ITV).^1^ In addition, interplay between the motion of the target and motion of the collimation can cause undesired dose distributions inside the PTV, especially for hypo‐fractionated treatments and cannot be accounted for with a margin.^2^

Respiratory motion is a primary source of intrafraction motion for treatment sites in the thorax and abdomen.^3^, ^4^ Motion management is recommended by the AAPM Task Group 76 (TG‐76) for respiratory motion >5 mm in any direction.^5^ Non‐ITV motion management techniques include gating and tracking, which both require precise knowledge of tumor location during treatment.^6^ Unfortunately, the precise location of the tumor during treatment is difficult to obtain. Target localization relies on internal or external surrogates, which can be determined based on optimal surface monitoring, radiofrequency beacons, kilovoltage (kV) x‐ray imaging, or magnetic resonance imaging (MRI).^3^, ^7^, ^8^

CyberKnife® (CK) uses the Synchrony® Respiratory Tracking system (Accuray Incorporated, Sunnyvale, CA), which combines external surrogate monitoring and x‐ray imaging of implanted fiducials to model and predict tumor motion due to respiration.^9^, ^10^, ^11^ In this system, the robotic movements of the CK delivery system are adapted in real time to compensate for motion.

**Fig. 1:**
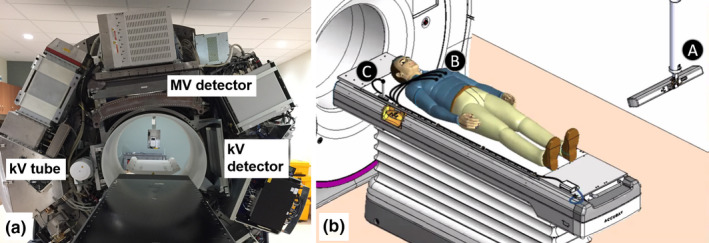
(a) A photograph of the Radixact system at UW‐Madison with the cover removed. The MV source is hidden by the couch in the photograph. (b) An illustration of the setup for a patient Synchrony treatment for respiratory motion. The light‐emitting diode (LED) camera (A) is mounted to the ceiling and monitors the position of LED's on the patient’s chest (B) and on the couch (C). Image courtesy of Accuray, Inc.

Radixact® (the next‐generation TomoTherapy® System; Accuray Incorporated, Sunnyvale, CA) is a helical tomotherapy radiation therapy delivery system capable of delivering conformal intensity‐modulated radiation therapy (IMRT).^12^ The continuous couch and gantry motion during treatment complicates conventional gating techniques. The Radixact contains an intrafraction motion management system called Synchrony®, which has been adapted from CK Synchrony.^13^ On the Radixact, an x‐ray tube and flat‐panel kV imager are offset 90° from the megavoltage (MV) imager and beam, shown in Fig. 1. The kV imaging subsystem is used to periodically localize the target during treatment (while the gantry is rotating). Two kV radiographs are separated in time to allow the gantry to rotate. Therefore, sequential monoscopic images provide delayed stereoscopic information. For monitoring respiratory motion, light‐emitting diodes (LEDs) are placed on the patients’ chest and identified with a camera mounted to the ceiling to provide the phase of respiration, shown in Fig. 1. The target can be localized with or without implanted fiducials near the target, but this work will only consider the fiducial‐based respiratory Synchrony option.

Schnarr et al. described the modeling of the target location based on the information from the kV radiographs and the external LEDs.^13^ The respiratory model is used to change the existing jaw locations and multi‐leaf collimator (MLC) leaf openings in real time during treatment. The model is updated every time new kV radiographs are acquired, without interrupting the treatment. Therefore, treatment time is the same for a Synchrony treatment as a conventional treatment unless the delivery has to be paused to acquire additional images to improve the motion model. The jaws compensate for target motion in the IEC‐Y (superior/inferior when head‐first supine) direction. Target motion in the IEC‐X (left/right) and IEC‐Z (anterior/posterior) directions is compensated by changes in the leaf opening positions. For example, when the gantry is at 0° (central axis along the IEC‐Z direction), motion in the IEC‐X direction is compensated by changing the leaf openings; motion in the IEC‐Z direction is not compensated since the target is moving along the line of the beam. Likewise, when the gantry is at 90°, the MLC leaf openings compensate for target motion in the IEC‐Z direction.

The dosimetric effect of a Synchrony‐enabled treatment for respiratory motion was studied by Schnarr et al., in which Synchrony tracked and compensated for linear respiratory‐mimicking motion, but for only one non‐clinical treatment plan for a cylindrical target.^13^ In addition, Chao et al. evaluated the accuracy of TomoTherapy dose calculations for Synchrony deliveries using film, but the motion was programmed *a priori* into the treatment plan instead of using real‐time tracking.^14^ Chen et al. performed acceptance testing of Synchrony deliveries,^15^ but these tests were limited to a single clinical delivery quality assurance (DQA) test for a respiratory with fiducial treatment. Akino et al. investigated the tracking accuracy of the CK Synchrony system and found that CK may suffer from large tracking errors in the presence of phase shifts between the surrogate and target motions.^16^ The effects of separate target and surrogate motion on tracking accuracies and dosimetric fidelity of Radixact Synchrony have not been investigated.

The current work investigates the Synchrony system on Radixact using real‐time tracking and modeling for realistic three‐dimensional (3D) respiratory motion and clinical IMRT plans. Two aims will be addressed: (a) evaluate the ability of the Synchrony system to accurately model and track motion of a phantom moving according to simulated respiratory motion, and (b) perform patient‐specific DQA to assess the deliverability of Synchrony plans correcting for undesired effects of intrafraction respiratory motion on helical tomotherapy treatments.

## 2 MATERIALS AND METHODS

Imaging datasets and treatment planning orders were selected for subjects with abdominal or thoracic tumors enrolled in an IRB‐approved study. Subject cases with lung, liver, and pancreas targets were selected to be replanned for delivery on a research Radixact system for this motion management study. Table 1 shows treatment information of each of the 13 subjects. All subjects had 4DCT scans as part of their clinical simulation, where they were instructed to breathe normally. The CTV was delineated on the maximum inspiratory breath hold (MIBH) image.

**Table 1:** Clinical cases, their prescriptions, tumor volumes, and beam‐on delivery times.

Case	Rx	PTV Volume (cc)	Plan delivery time (s)
Lung 1	2 Gy × 30	83.1	126
Lung 2	10 Gy ×5	4.1	677
Lung 3	10 Gy ×5	2.2	694
Lung 4	9 Gy ×5	6.2	536
Lung 5	2.25 Gy ×20	544.3	252
Liver 1	8 Gy ×5	113.2	462
Liver 2	2.5 Gy ×18	114.0	253
Liver 3	3.87 Gy ×15	498.8	556
Liver 4	8 Gy ×5	140.5	568
Pancreas 1	6 Gy ×5	22.8	287
Pancreas 2	6 Gy × 5	43.7	289
Pancreas 3	2 Gy ×25	30.9	208
Pancreas 4	3 Gy ×10	195.1	246

### 2.A. Motion traces

Motion traces were generated uniquely for each subject in MATLAB® (MathWorks, Inc., Natick, MA). Characteristics of the motion traces used in this work for the 13 subjects are shown in Table 2. Motion was modeled using an equation proposed by Lujan et al.,

(1) $$A_{X,Y,Z,S}\left(t\right)=A_{X,Y,Z,S}\cdot \sin^{2n}\left(\frac{\pi t}{T}‐\varphi_{Z,S}\right),$$

where *A* is the amplitude, *t* is time, *T* is the period, φ is the starting phase in radians, and *n* is a fitting parameter to model more time spent at exhale than inhale.^17^ Equation (1) was used to separately model the X, Y, and Z motion of the target and the Z motion of the patient's chest, or the surrogate (S). The period and amplitude for each respiration were randomly sampled from a normal distribution. The mean and standard deviations of these normal distributions were chosen using values that are typical of patient respirations observed in the literature, such as a mean period between 3 and 5 s.^3^, ^4^ The period and amplitude of subsequent respirations were smoothed using a moving average filter to prohibit sudden changes in these parameters between respirations. Hysteresis in the sagittal plane was modeled by a phase shift in the Z direction (φ_Z_) relative to the Y direction, which was constant throughout a given subject’s motion. Phase shifts between target motion and surrogate motion were modeled by φ_S_, which was also constant throughout a given subject’s motion. A positive phase shift for the S or Z motion indicates that the motion in that direction is delayed. Shifts are specified in terms of percent: a shift of π/2 is denoted a 50% phase shift. The fitting parameter (*n*) was chosen to range from 1 to 3 in this work based on the observations of Seppenwoolde et al.^18^. A shift in baseline position — modeled by a low‐frequency, low‐amplitude cosine — was incorporated into the traces since these shifts have been observed to have the greatest effect on helical tomotherapy treatments.^19^ The baseline shifts throughout treatment ranged from 0 to 2.5 mm.

**Table 2:** Characteristics of the motion traces generated for each subject case. The parameters are in reference to Eq. (1). The fitting parameter (*n*) was 2 for all cases in this table. The X and Y directions were always in phase (φ_X_ = 0). The RMS displacement from the origin is a metric used to describe the 3D magnitude of motion from the phantom origin location (where it was registered). With motion tracking turned off, δ_RMS_ for each case in Table 3 would be equal to this value.

	$\overset{‐}{A_{Y}}$ (mm)	$\overset{‐}{A_{Z}}$ (mm)	$\overset{‐}{A_{X}}$ (mm)	RMS displacement from origin (mm)	$\overset{‐}{T}$ (s)	φ_Z _(%)
Lung 1	16	10	6	6.9	5	20
Lung 2	5	10	9	5.4	5	−10
Lung 3	7	14	13	7.4	5	−20
Lung 4	12	13	13	8.0	5	0
Lung 5	10	6	2	5.6	5	0
Liver 1	9	2	1	3.4	3	−50
Liver 2	11	14	5	6.8	4	−60
Liver 3	16	5	1	6.1	4	10
Liver 4	18	16	6	9.4	3	0
Pancreas 1	13	3	1	5.4	3	20
Pancreas 2	14	11	5	6.8	5	−50
Pancreas 3	14	3	1	5.3	4	−10
Pancreas 4	10	6	6	5.1	4	−20

### 2.B. Treatment planning

For each subject, a helical tomotherapy plan was generated in the Accuray Precision® Treatment Planning System (TPS) according to objectives intended for a gated delivery. A PTV was created by adding a tight, 3 mm margin around the CTV, as defined on the MIBH scan. This margin is based on what is routinely used for gated delivery at our institution. As with gated delivery techniques, tracking techniques such as Synchrony are implemented to decrease margins, reduce motion interplay, and negate the need for an ITV.^6^ Therefore, this study intentionally avoided the use of an ITV and used margins that would have been used for gated delivery. The optimization and dose calculation for all plans were performed on the static MIBH dataset. Finally, a DQA treatment plan was generated by recalculating the optimized plan on the measurement phantom dataset.

The maximum width projected to isocenter the jaws can open to is 5 cm, but this setting is not available for Synchrony since there would be little dynamic range for IEC‐Y (superior/inferior) plan modulation. Subjects can only be treated with the 2.5 cm jaw settings if their motion in the IEC‐Y direction is approximately <2.5 cm peak‐to‐peak and the target is aligned to the mean position. There is no mechanical limit on amplitude of motion in the IEC‐X and IEC‐Z directions (other than extreme off‐axis targets), as motion in these directions are compensated by MLC leaf openings. The 2.5 cm jaw setting was used for all plans in this study.

### 2.C. Validation measurements

**Fig. 2:**
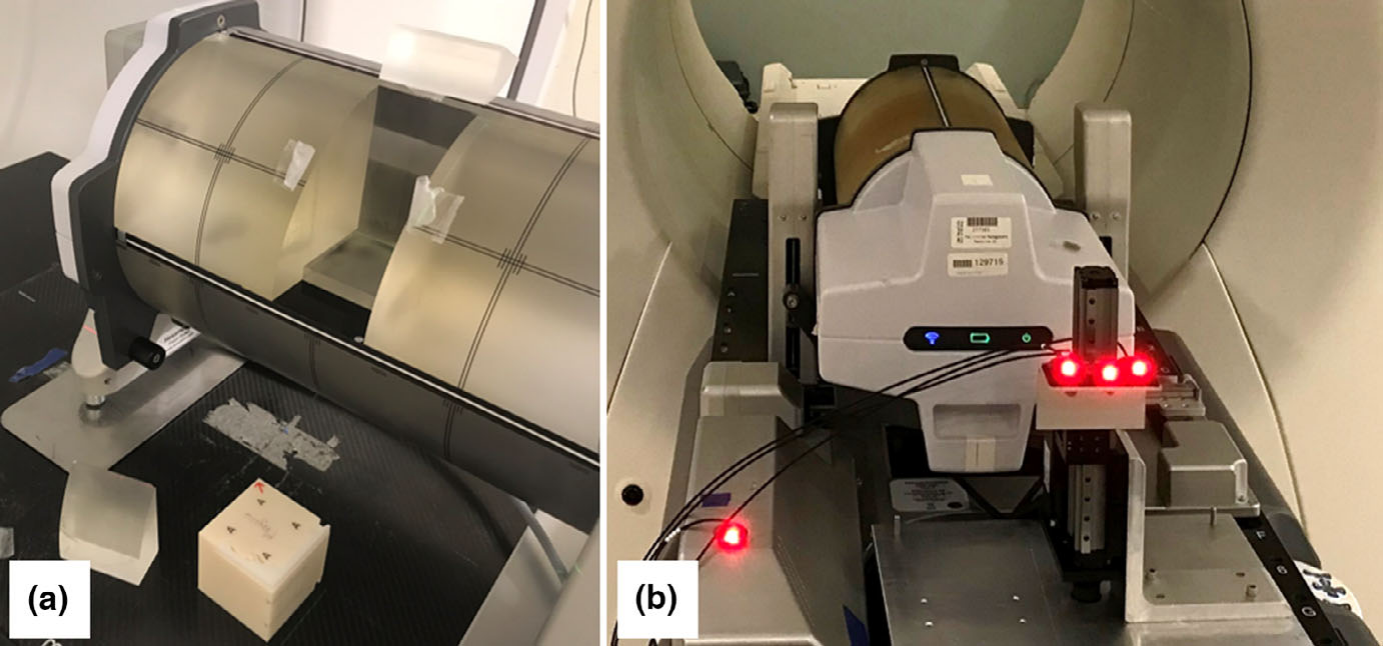
(a) The modified Phantom+ with the ball‐cube removed. The ball‐cube holds fiducials. (b) The Phantom+ on the Hexamotion stage. The three patient light‐emitting diodes (LEDs) were either placed on the separate surrogate stage (shown here), or on the Phantom+, and one couch LED was placed on the couch or a static object on the couch.

Delivery validation of these clinical patient plans was performed using a customized Phantom+ and a Hexamotion® stage (ScandiDos Inc., Uppsala, Sweden), shown in Fig. 2. The Hexamotion stage provided 3D translational movements described by the generated motion traces. The Phantom+ was modified to house a CyberKnife “ball‐cube” insert with embedded fiducials (Fig. 2). These fiducials are imaged with the kV radiographs and are used as a surrogate of target position.

Two LED placement locations were used in this work. For the 13 subject cases, the LEDs were placed on the Phantom+ itself. For one subject case (Lung 5), additional investigations were performed with the LEDs placed on a separate, 1D surrogate stage in front of the Phantom+, shown in Fig. 2. This setup was used to explore the effect of varying the relationship between the surrogate LED motion and the internal target motion, as described by Akino et al.^16^. The motion of the surrogate stage replicates motion of the patient’s chest in the IEC‐Z direction, which may be different from the motion of the internal tumor, often majorly in the IEC‐Y direction. For all Synchrony deliveries, one LED is placed on the couch (or a static object on the couch) and is used by the algorithm as a baseline. For the surrogate stage investigations, the dosimetric delivery was left unchanged and the effects of changing various parameters on the tracking accuracy were assessed individually: LEDs on Phantom+ vs LEDs on surrogate, amplitude of surrogate motion (*A_S_*), phase of the surrogate with respect to the target phase (φ_S_), and the fitting parameter (*n*) of the surrogate and target motion.

Three scenarios of the DQA treatment plan were delivered separately for each of the 13 subjects. A moving phantom is denoted M1 and a stationary phantom is denoted M0. Likewise, the scenario with Synchrony enabled is denoted S1 and without Synchrony enabled is denoted S0. The first scenario is M0S0, or a static phantom and no Synchrony. This represents the ideal, but unrealistic, dose delivery if the target was not moving during treatment. The second scenario is M1S0, or no Synchrony but a moving phantom. This represents conventional tomotherapy delivery conditions with intrafraction motion but with a tight margin that would not have been used without motion management. Comparison of the M0S0 and M1S0 scenarios investigates the effect of intrafraction motion on a conventional helical tomotherapy treatment, which has been done extensively in the literature^19^, ^20^, ^21^, ^22^, ^23^ and therefore was not the focus of this work. For the third scenario, M1S1, the phantom was moving, and motion Synchrony was enabled. The focus of this work is to compare the M0S0 and M1S1 plans, but without comparisons with M1S0 the effect of using Synchrony is unclear. Therefore, the M1S0 case was used to benchmark the impact that the motion had upon the static plan.

For the Synchrony deliveries, the internal fiducials were identified on the MVCT scan during treatment setup where the phantom was aligned to the planning image. The user chooses the angles and the number of the kV radiographs, which ranged from 3 to 5 per gantry rotation. The imaging parameters were the preset values for a medium‐sized thorax image: 1.0 mAs and 120 kV. The number of images acquired during a Synchrony treatment is a function of the gantry rotation speed, treatment time, and number of images per gantry rotation angle.

Synchrony models the offset of the phantom with respect to the registered position of the phantom and uses this information to adjust the treatment parameters. The first aim of this work was accomplished by comparing a log file of the modeled motion at every point during the treatment to the known motion trace supplied to the Hexamotion stage. The log file is generated by the Radixact system after each Synchrony delivery. The resolution in time of the log file was determined by the LED camera acquisition rate, which is about one per 12.5 ms. The phantom motion trace was resampled to match the LED camera acquisition rate. The RMS error between the Synchrony‐predicted motion and the actual phantom motion was calculated using Eq. (2), where ${\vec{r}}_{S}\left(t\right)$ are the Synchrony‐predicted positions as a function of time and ${\vec{r}}_{P}\left(t\right)$ are the motion trace positions as a function of time that were supplied to the Hexamotion stage. In addition, tracking accuracies were also measured using the probability of observing a particular error during treatment, denoted δ_%_. For example, a value of 1 mm for δ_95%_ would indicate that for 95% of the treatment time, the error between phantom motion and the tracked motion was 1 mm or less.

(2) $$\delta_{\mathrm{RMS}}=\sqrt{\frac{\sum_{i=1}^{N}{\left|{\vec{r}}_{S}\left(t\right)‐{\vec{r}}_{P}\left(t\right)\right|}^{2}}{N}}$$

The three dose distributions (M0S0, M1S0, M1S1) measured with the Phantom+ and the planned dose distribution were intercompared. The 3D planned dose was calculated on the static MIBH dataset and exported from the Precision TPS into the Delta4 software. The Phantom+ has two planar, orthogonal diode grids providing two two‐dimensional (2D) dose arrays for each measurement. The central region of the Phantom+ has a diode spacing of 2.5 mm and the peripheral region has a diode spacing of 5 mm. Delivery was planned such that the high dose region was centered over the high‐resolution portion of the Phantom+ diode array.

The dose initially measured with the Phantom+ during the M1S1 delivery includes a response from the kV radiographs acquired during the measurements. The diodes tend to over‐respond to the kV photons relative to the 6 MV photons. Also, the dose from the kV images is not intended to contribute to the therapeutic dose. Since there are no kV images acquired for the M0S0 or M1S0 deliveries, only the M1S1 deliveries record this extra dose in the DQA measurements. Therefore, additional measurements were performed to subtract out the diode response to the kV images. This was done by redelivering the M1S1 plans using only the kV images (no MV beam) and subtracting this dose distribution out of the measured M1S1 dose distribution for each plan.

The first type of dosimetric analysis was to compare each of the measured doses to the planned dose. This represents the traditional goal of phantom DQA measurements: how well does the measured dose match the planned dose. The second type of dosimetric analysis was to compare the three measured dose distributions to each other. More specifically, the M1S0 and M1S1 measured doses were compared to the M0S0 dose using metrics of gamma pass rate and median dose difference. This was performed in order to uncouple the deliverability and measurement device errors of the static M0S0 plan from the M1S0 and M1S1 plans. If the M0S0 plan differs from the planned dose, it is expected that this difference will be apparent in the other measured dose distributions as well. For gamma analysis, criteria recommended by the AAPM Task Group 218 (TG‐218) for evaluating IMRT plans were used: global 3%/2 mm for points above 10% of the maximum dose.^24^ The median dose difference was intended to quantify dosimetric differences inside the target region, therefore a threshold was applied considering points above 50% of the maximum dose. All dose comparison metrics were calculated using the Delta4 software. One‐dimensional profiles in the X, Y, and Z directions were extracted from the Delta4 software to analyze the shape of the dose distributions.

## 3 RESULTS

### 3.A. Motion Tracking

**Fig. 3:**
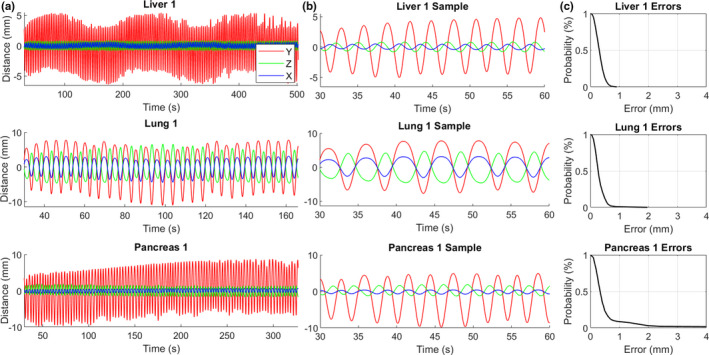
(a) Examples of treatment‐length phantom motion traces for three subject cases. (b) 30‐s samples of the phantom motion traces. (c) Cumulative tracking error plots showing the probability of observing a tracking error greater than the specified value throughout the treatment.

Table 3 shows tracking error statistics between the Synchrony‐predicted motion and the phantom motion for each of the 13 M1S1 subject cases with the LEDs on the Phantom+. The ratio of respiratory period to the average time between the kV radiographs, or “images per respiration,” varied from 0.4 to 1.6, as shown in Table 3. The values of δ_RMS_ were 1.5 mm or less for all cases and the values of δ_95%_ were <3.0 mm for all cases. Motion traces and tracking error plots for three example subject cases are shown in Fig. 3.

**Table 3:** Tracking results for the M1S1 cases with the LEDs on the Phantom+. Motion parameters for each case are provided in Table 2. The average number of images per respiration is dependent on planning parameters such as the gantry rotation period and the number of kV images per rotation.

	Average # of kV images per respiration	δ_RMS_ (mm)	δ_95%_ (mm)	δ_50%_ (mm)
Lung 1	1.6	0.4	0.6	0.3
Lung 2	0.6	0.7	1.4	0.5
Lung 3	0.6	0.6	1.1	0.5
Lung 4	1.6	0.6	1.1	0.4
Lung 5	1.6	0.3	0.3	0.1
Liver 1	1.3	0.4	0.6	0.3
Liver 2	0.8	1.5	2.9	1.1
Liver 3	0.5	0.6	1.1	0.4
Liver 4	0.4	0.8	1.6	0.5
Pancreas 1	0.6	1.1	1.7	0.3
Pancreas 2	1.2	1.0	1.8	0.8
Pancreas 3	0.9	0.5	0.9	0.3
Pancreas 4	0.7	0.5	1.0	0.4

**Fig. 4:**
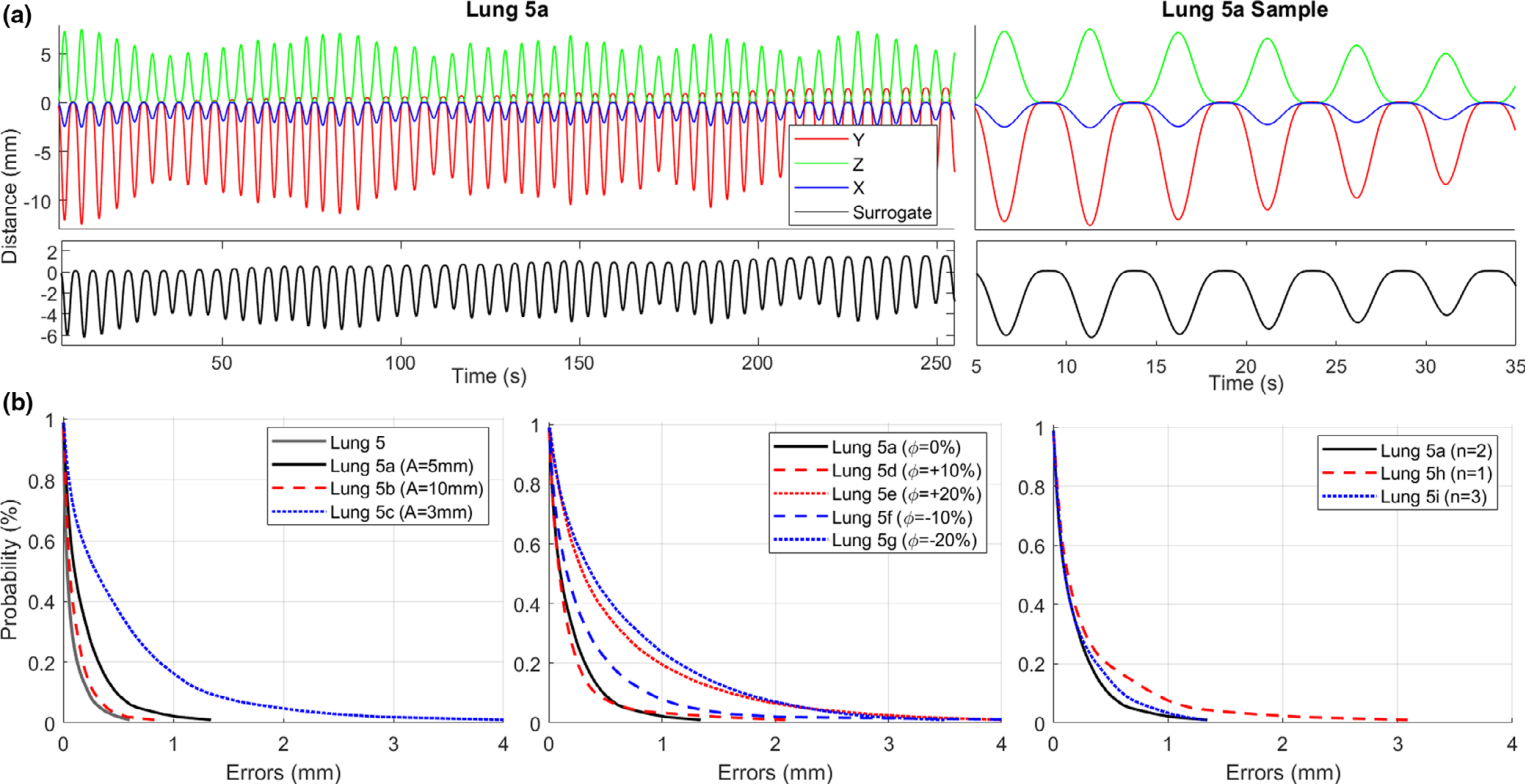
(a) Full‐treatment and 30‐second sample phantom motion trace for Lung 5a in the X, Y, Z, and surrogate directions. The X, Y, Z motion of the target was not changed for cases Lung 5b‐Lung 5i (other than fitting parameter change for Lung 5h and Lung 5i). (b) Cumulative tracking error plots showing the probability of observing a tracking error greater than the specified value throughout the treatment for various cases.

Table 4 shows tracking error statistics between the Synchrony‐predicted motion and the phantom motion for each of the cases of Lung 5 with the LEDs on the Phantom+ or on the surrogate stage. The original case with the LEDs on the Phantom+ is termed “Lung 5” and the additional cases with the LEDs on the surrogate stage were termed “Lung 5a”‐“Lung5i.” For Lung 5b‐Lung 5i, only one parameter was changed relative to Lung 5a at a time. The motion parameters of Lung 5a and the changes of the subsequent cases are shown in Table 4. Figure 4 shows target and surrogate motion traces for Lung 5a and tracking error plots for cases Lung 5‐Lung 5i. The values of δ*_RMS_* were 1.0 mm or less and the values of δ_95%_ were <2.5 mm for all Lung 5 cases. The cases with the best tracking accuracies were with the LEDs on the Phantom+ (Lung 5) and with a large surrogate amplitude (Lung 5b). The cases with the worst tracking accuracies were with phase shifts of ±20% (Lung 5e and Lung 5g) and with small surrogate amplitude (Lung 5c). Accuracies and dosimetric outcomes were largely unchanged with changes in the fitting parameter (*n*).

**Table 4:** Dosimetric and tracking analysis for the various LED motions for the Lung 5 case with separate LED and phantom motion. The parameters of the Lung 5a case were used as the base parameters: mean surrogate amplitude ($\overset{‐}{A_{S}}$) of 5 mm, phase shift (φ_S_) of 0%, fitting parameter (*n*) of 2, and mean period ($\overset{‐}{T}$) of 5 s. For Lung 5b‐5i, only one parameter was changed relative to Lung 5a. Gamma pass rates are for the M1S1 case. The pass rates for the M1S0 case for Lung 5 are in Tables 5 and 6.

Case	LED location	Parameter change from Lung 5a	γ pass: 3%/2 mm/10%T (to plan)	γ pass: 3%/2 mm/10%T (to M0S0)	δ_RMS_ (mm)	δ_95%_ (mm)
Lung 5	Phantom+	–	100.0	100.0	0.3	0.3
Lung 5a	Surrogate	None	96.7	99.9	0.5	0.7
Lung 5b	Surrogate	$\bar{A_{S}}$ = 10 mm	99.8	100.0	0.5	0.4
Lung 5c	Surrogate	$\bar{A_{S}}$ = 3 mm	94.5	99.4	0.8	2.0
Lung 5d	Surrogate	φ_S_ = +10% ^a^	99.8	100.0	0.5	0.7
Lung 5e	Surrogate	φ_S_ = +20%	97.5	99.5	0.8	2.3
Lung 5f	Surrogate	φ_S_ = −10%	99.3	100.0	0.6	1.3
Lung 5g	Surrogate	φ_S_ = −20%	95.0	99.2	0.8	2.3
Lung 5h	Surrogate	*n* = 1^b^	98.9	100.0	0.6	1.2
Lung 5i	Surrogate	*n* = 3^b^	98.3	100.0	0.5	0.9

^a^ A 10% shift of a 5 s period is 0.25 s.

^b^ The same fitting parameter (*n*) was used in Eq. (1) for both the LED and target motion when the fitting parameter was changed. The amplitude and period of motion of both the LED and target motion were unchanged when this parameter was changed.

### 3.B. Dosimetry

Dosimetric analysis between each of the three measured doses and the planned dose for the 13 cases with LEDs on the Phantom+ is shown in Table 5. All the M0S0 and M1S1 measured dose distributions and only two of the M1S0 measured dose distributions had gamma pass rates (3%/2 mm/10%T) above the universal action limit (90%) as recommended by TG‐218.^24^ All the M0S0 and M1S1 measured dose distributions and eight of the M1S0 measured dose distributions had a median dose difference (50% threshold) compared to the planned dose within ±3%.

**Table 5:** Dosimetric analysis of the three measured doses compared to the planned dose. The LEDs were placed on the Phantom+ for these cases. Motion parameters for each case are provided in Table 2. Gamma analysis and median dose differences are relative to the global maximum planned dose. A positive median dose difference indicates the measured dose was greater than the planned dose. Only points above the specified dose threshold were considered.

	γ pass: 3%/2 mm/10%T			Med dose diff: 50%T (%)		
	M0S0	M1S0	M1S1	M0S0	M1S0	M1S1
Lung 1	97.3	61.3	96.5	1.8	−2.2	1.3
Lung 2	100	98.1	100	−1.1	−2.6	−1.2
Lung 3	100	86.2	100	0.1	−7.7	−2.1
Lung 4	99.6	83.6	98.8	0.7	−7.9	−1.1
Lung 5	100	83.4	100	−0.3	−2.5	−0.8
Liver 1	94.5	83.1	90.7	2.3	1.3	2.6
Liver 2	99.8	44	95.1	1.5	−4.2	0.6
Liver 3	100	78.7	99.9	−0.8	−2.8	−0.8
Liver 4	100	67.8	96.7	−1.6	−2.7	−2.2
Pancreas 1	99.8	87.9	99.8	0.8	−4.0	0.2
Pancreas 2	98.3	73.3	90.1	1.8	−4.3	0.9
Pancreas 3	100	70.1	98.9	−1.0	−2.4	−1.4
Pancreas 4	100	95.9	97.9	0.3	0.2	0.2
Mean (Std.)	99.2 (1.6)	78.0 (14.7)	97.3 (3.4)	0.3 (1.3)	−3.2 (2.6)	−0.3 (1.4)

Table 6 shows comparisons of the M1S0 and M1S1 measured doses to the M0S0 measured dose. The gamma pass rate (3%/2 mm, 10% threshold) relative to the M0S0 dose was >90% for all the M1S1 cases and four of the M1S0 cases. The gamma pass rate was >99% for 10 of 13 of the M1S1 cases. The median dose difference (50% threshold) between M1S0 and M0S0 was negative for all subject cases (average of −3.6%), indicating the target may be underdosed from the M1S0 plans. The median dose difference between M1S1 and M0S0 was negative for 9 of 13 subject cases (average of −0.6), indicating there was a minor underdosing above 50% dose for the M1S1 cases with respect to the M0S0 cases.

**Table 6:** Dosimetric analysis of the measured doses with motion (M1S0 and M1S1) compared to the static measured dose (M0S0). The LEDs were placed on the Phantom+ for these cases. Motion parameters for each case are provided in Table 2. A positive median dose difference indicates the M1S1 or M1S0 dose was greater than the M0S0 dose.

	γ pass: 3%/2 mm/10%T		Med dose diff: 50%T (%)	
	M1S0	M1S1	M1S0	M1S1
Lung 1	63.4	99.6	−4.6	−0.5
Lung 2	98.8	100.0	−1.9	−1.0
Lung 3	94.2	100.0	−7.9	−1.6
Lung 4	88.9	100.0	−8.3	−1.7
Lung 5	86.6	100.0	−1.1	−0.5
Liver 1	97.8	100.0	−1.3	0.1
Liver 2	41.7	99.2	−6.0	−0.7
Liver 3	87.2	100.0	−2.0	0.2
Liver 4	82.8	100.0	−0.7	−0.5
Pancreas 1	88.9	100.0	−5.0	−0.4
Pancreas 2	68.6	94.0	−5.9	−0.5
Pancreas 3	76.1	97.9	−1.3	−0.1
Pancreas 4	99.6	99.4	−0.2	0.0
Mean (Std.)	82.7 (16.6)	99.2 (1.7)	−3.6 (2.8)	−0.6 (0.6)

Two‐tailed paired T‐tests (n = 13) were performed for the data shown in Table 6, comparing the M1S0 and M1S1 dose distributions to the M0S0 dose distributions. There was evidence (*P* = 0.003) suggesting a difference between the gamma pass rate between the M1S0 and M0S0 plans and the gamma pass rate between the M1S1 and M0S0 plans. There was also evidence (*P* < 0.001) suggesting a difference between the median dose difference between the M1S0 and M0S0 plans and the median dose difference between the M1S1 and M0S0 plans.

**Fig. 5:**
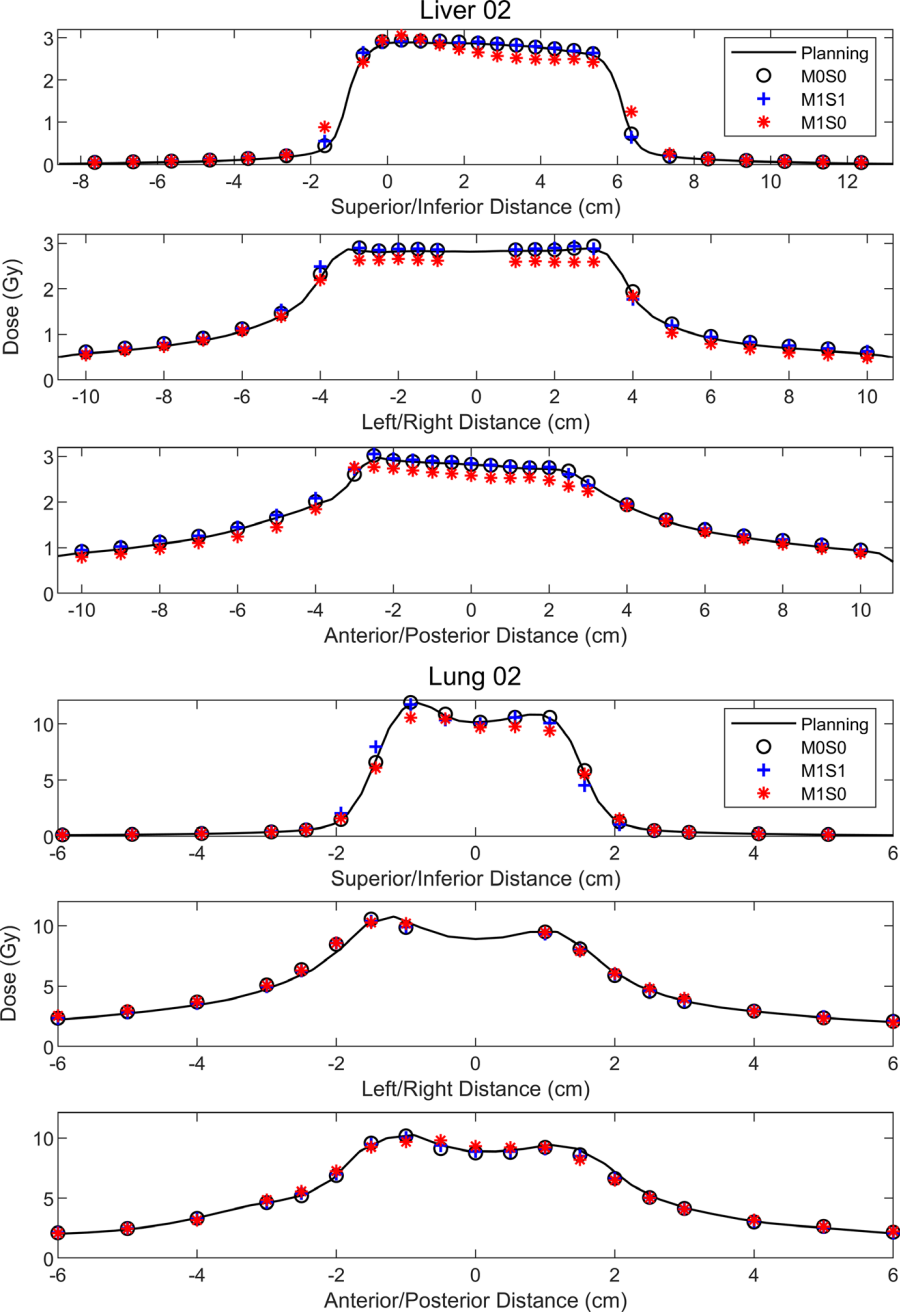
Profile analysis for Liver 2 and Lung 2. Profiles were acquired through diodes passing through the geometric center of the Phantom+. Liver 2 was chosen to demonstrate effects of interplay and dose blurring for the M1S0 plan, that are not present for the M1S1 plan. Lung 2 was chosen to demonstrate that although the gamma pass rates for the M1S0 delivery were high (>98% in Table 5), the shape of the dose distribution for M1S0 did not match the planned or static measured distributions as well as for M1S1.

Figure 5 shows example profile comparisons between the measured and planned doses for the Liver 2 and Lung 2 cases. Profiles are acquired through the geometric center of the Phantom+ in the X, Y, and Z directions.

## 4 DISCUSSION

### 4.A Motion tracking

**Fig. 6:**
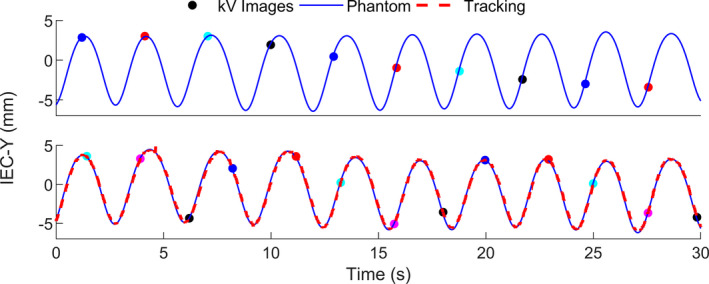
30‐s sample of Synchrony‐modeled motion (“Tracking”) vs phantom motion in the IEC‐Y direction for Liver 1 with four images per gantry rotation (top) and five images per gantry rotation (bottom). Multicolored points indicate the phase at times which the kV images were acquired (four colors in the top and five colors in the bottom). When imaging with four images per gantry rotation, the imaging frequency was found to alias with the breathing frequency and the model was not accurately built (hence there is no tracking curve in the top image).

If the ratio of the time between images to the respiratory period is one, that means that exactly one image is acquired per respiration. As a result of this, aliasing can occur between the kV images and the respirations, that is, the images may be acquired only at one phase of the breathing cycle. This can lead to uncertainties in target position during the other phases of the breathing cycle. For Liver 1, this was initially observed when using four imaging angles per gantry rotation, shown in Fig. 6. The average breathing period was 3 s and the gantry rotation period was 11.8 s, therefore the number of images per respiration was approximately 1.0. The model was not able to be accurately built with these parameters. The plan was changed to have five images per gantry rotation (~1.3 images per respiration), and the M1S1 plan was successfully delivered. Figure 6 shows that the kV images sampled all phases of respiration more evenly with five images per gantry rotation than four. The number and angles of kV images per gantry rotation can be chosen prior to the treatment and can be modified during the treatment if aliasing is observed. Breath‐coaching could be used to avoid respiratory frequencies aliasing with the imaging frequencies.

In this work, the number of images per respiration was not found to be correlated with tracking accuracy as long as this value was not near unity. For example, this value was the smallest at 0.4 for Liver 4, and the RMS error between the Synchrony trace and the phantom trace was 0.8 mm. Images were acquired only once every 2.5 respirations, but this work indicates that as long as respiration is regular during this time, the model will stay relatively constant and does not require more frequent updating. Future work will investigate the limits of imaging frequency and accuracy of the resulting model.

With the LEDs placed on the surrogate stage instead of on the Phantom+, the tracking accuracies were slightly worse. However, the tracking accuracies for all cases of Lung 5 were similar to tracking accuracies reported for the CK Synchrony motion tracking system. For example, for similar tracking experiments on CK Synchrony, Akino *et al*. reported values of δ_95%_ to be as large as 2.8 mm for irregular respiratory motion with the surrogate and target in‐phase^16^ and Inoue et al. reported values of δ_95%_ ranging from 1.0 to 3.5 mm.^25^ Akino et al. found extremely large tracking errors when the surrogate and the target were out of phase, such as δ_95%_ >9 mm for a surrogate phase of +15% and δ_95%_ >6 mm for a surrogate phase of +10%. The largest values of δ_95%_ in the current work were 2.9 mm for the Liver 1 case (Table 3) and 2.3 mm for the Lung 5e and Lung 5g cases (Table 4), which had phase shifts larger than that investigated by Akino et al. The fundamental motion model used by the Radixact Synchrony and CK Synchrony are different as described by Schnarr et al., therefore it is not expected that these systems would have the same sensitivities to surrogate phase shifts.^13^

### 4.B Dosimetry

No obvious correlation was observed between the tracking errors and the dosimetric outcome of the plans. For example, the RMS error between the tracked and modeled motion traces was largest for the Liver 2 case at 1.5 mm (Table 3), but the gamma pass rates for the M1S1 dose compared to the planned dose (Table 5) and the M0S0 dose (Table 6) were 95.1% and 99.2%, respectively. In addition, there was no obvious correlation between treatment plan parameters (gantry period, kV image frequency, etc.) and the dosimetric fidelity of the cases. To explore this, additional cases would need to be included while controlling for variable changes, which was outside the scope of this work.

The gamma pass rates compared to the planned dose for the cases with the LEDs on the surrogate stage were all >90%, shown in Table 4. In addition, the gamma pass rates compared to the M0S0 measured dose were all >99% with the LEDs on the surrogate stage. This indicates that dosimetric deliverability of the Lung 5 plan was largely unchanged with varying surrogate motion parameters. Although independent surrogate/target motion was only tested for one subject case, all parameters in the motion model were changed independently and DQA was measured for each. The lowest gamma pass rate compared to the planned dose was observed for the case with a small surrogate amplitude (Lung 5c) at 94.5%. However, this plan is still above the action limit per TG‐218 recommendations and is much larger than the pass rate (86.6%) if no motion management was used.

The profiles in Fig. 5 demonstrate the potential dose blurring and motion interplay when no motion management is used. For both Liver 2 and Lung 2, the penumbra of the M1S0 dose distribution in the superior/inferior direction is wider than that of the M1S1 or M0S0 dose distributions. In addition, motion interplay is observed in the superior/inferior M1S0 profile of Liver 2, as the shape of the dose distribution was largely changed. If motion blurring alone was occurring, the M1S0 dose is expected to be a weighted average of the M0S0 dose over the path of motion, that is, the dose at any point would be convolved by the same response function determined by the path of motion. Although motion interplay was not observed in the M1S0 profiles for all the subject cases in this work, when there was motion interplay in the M1S0 profiles, it was not observed in the M1S1 profiles. No interplay was observed in the M1S1 profiles for any subject cases. Although ITV‐based motion management strategies can account for dose blurring due to motion (at the expense of irradiating more normal tissue), it cannot adequately manage interplay between target motion and the delivery like active motion management such as Synchrony.^2^

It is important to note that if motion management is used when it was not actually necessary, this does not introduce more errors to the delivery that would not have been introduced without motion management. There were cases (Lung 2 and Pancreas 4) in which no large difference in gamma pass rate was observed when Synchrony was used or not used. For the M1S0 and M1S1 cases for both Lung 2 and Pancreas 4, the gamma pass rates compared to the M0S0 dose were all greater than 98% (Table 6), suggesting motion management would not have been needed in these cases for a gamma‐based DQA.

The profiles for Lung 2 were included in Fig. 5 to show that even though gamma pass rates were high without motion management, dose blurring can be observed in the M1S0 profiles in the superior/inferior and anterior/posterior directions which is not observed in the M1S1 profiles. It is unclear whether these changes in the profiles necessitate motion management, but it is clear that when motion management was used, the resulting profiles more closely resembled the static profiles.

Population statistics were intended to indicate whether phantom motion has a significant impact on the dose distribution when compared to the M0S0 dose and whether motion synchronization corrects for this. Although this is a small subset of cases (n = 13), there was evidence suggesting a difference between using Synchrony (M1S1) and not using Synchrony (M1S0) on the dosimetric similarity to the static measured dose (M0S0) for the metrics shown in Table 6. Motion Synchrony did not have a large benefit for every subject in this work, but these statistics indicated that for these subjects and metrics, there was an overall significant improvement when Synchrony was used compared to when it was not used.

## 5 CONCLUSION

The motion Synchrony system on the Radixact was able to track and synchronize the delivery of radiation with realistic 3D respiratory motion of a phantom for clinical helical tomotherapy treatments. When motion Synchrony was used with a moving phantom, the measured dose distribution more closely matched the planned dose. The impact of motion on the treatments was found to vary from subject to subject, but overall, there was evidence suggesting significant improvement in agreement with the static dose distribution when using Synchrony. In addition, motion interplay and dose blurring effects were not observed with Synchrony enabled. The sensitivities of CK Synchrony to surrogate phase shifts reported in the literature^16^ were not observed for the Radixact Synchrony system. This work provided evidence that Synchrony reduces effects of intrafraction respiratory motion that are not accounted for using the current, ITV‐based motion management strategy for helical tomotherapy plans.

## CONFLICT OF INTEREST

Michael Kissick is an employed by and has ownership interests in Accuray Inc.
