# Erratum: “Evaluation of radixact motion synchrony for 3D respiratory motion: Modeling accuracy and dosimetric fidelity”

**DOI:** 10.1002/acm2.13805

**Published:** 2023-01-20

In Ferris et al.,^1^ the corresponding author has requested to update the Conflict of Interest. The Conflict of Interest should be updated as below.

Michael Kissick is employed by and has ownership interest in Accuray, Inc. John E Bayouth has ownership interest in MR guidance, LLC, which has business activity with a company that utilizes respiratory motion management (ViewRay, Inc.). The remaining authors have no conflicts of interest to disclose.
